# Serum Cytokine Profiles in Children with Crohn's Disease

**DOI:** 10.1155/2016/7420127

**Published:** 2016-12-13

**Authors:** Ekaterina Vasilyeva, Sayar Abdulkhakov, Georgi Cherepnev, Ekaterina Martynova, Irina Mayanskaya, Alina Valeeva, Rustam Abdulkhakov, Dilyara Safina, Svetlana Khaiboullina, Albert Rizvanov

**Affiliations:** ^1^Volga Region Federal Medical Research Center of the Ministry of Public Health, Nizhny Novgorod, Russia; ^2^Institute of Fundamental Medicine and Biology, Kazan Federal University, Kazan, Russia; ^3^Kazan Federal University Clinic, Kazan, Russia; ^4^Department of Clinical Laboratory Diagnostics, Kazan State Medical Academy, Kazan, Russia; ^5^Kazan State Medical University, Kazan, Russia; ^6^Department of Microbiology and Immunology, University of Nevada Reno, Reno, NV, USA

## Abstract

Crohn's disease (CD) is a chronic inflammatory bowel disease that can be diagnosed at any age. There are two major patient groups based on diagnosis of this disease, before or after the age of 20 (juvenile/adolescent or adult), with disease progression in adults usually milder than in juvenile CD patients. Immune mechanisms have been suggested to play an important role in CD pathogenesis, with cytokines governing the development of the immune response. Upregulation of inflammatory cytokines in serum of juvenile and adult CD patients has been documented; still little is known about age-dependent differences in serum cytokine profiles of CD patients. We applied multiplex technology to analyze serum levels of 12 cytokines in juveniles and adults. We show that during the acute stage of the disease all CD patients have high serum levels of CXCL10, which remains upregulated during remission. Increased serum levels of TNF-*α* and IL-6 during the acute stage was characteristic of juvenile CD patients, whereas adult CD patients had upregulated levels of GM-CSF and IFN-*γ*. Taken together, these results demonstrate age-dependent differences in cytokine profiles, which may affect the pathogenesis of CD in patients at different ages of disease onset.

## 1. Introduction

Crohn's disease (CD) is a form of inflammatory bowel disease (IBD) affecting the entire gastrointestinal (GI) tract [1–4]. CD is characterized by transmural inflammation of GI wall in a noncontiguous pattern anywhere from the mouth to the anus [5, 6]. Little is known about the etiology. It is believed that, in genetically predisposed individuals, interaction between local microbiota and the intestinal mucosa can cause chronic inflammation and ulceration [7–9]. A key pathological feature of CD is a granulomatous inflammatory response, characterized by the presence of epithelioid and multinucleated giant cells as well as macrophage infiltration [10, 11]. It is believed that the onset and development of the inflammatory reaction depend on persistence of an inciting agent, resulting in a chronic inflammatory milieu and perpetuating a complex immune reaction subsequent to the local granulomatous response. Persistent inflammation and immune activation are believed to be the leading causes of tissue necrosis and fibrosis.

Clinically CD is characterized by reoccurring episodes that are typical of IBD [12]. Newly diagnosed CD usually presents with diarrhea, abdominal pain, fever, fatigue, stomatitis, and weight loss [12]. In more advanced cases, strictures and fistulas may develop, changing the clinical presentation to include severe abdominal pain, distension, bloating, vomiting, perianal fistulae, and abscesses. CD can be diagnosed at any age; however disease progression and clinical presentation frequently differ in young and adult patients [13]. CD in children and adolescents tends to have a more severe clinical manifestation often with development of strictures or fistulae [1, 3, 14]. In contrast, later onset of CD is often characterized by a milder course with a lower frequency of complications [13, 14]. Furthermore, it has been shown that the childhood-onset CD is more likely to require immunosuppressive therapy and has a higher frequency of surgery as compared to CD diagnosed in adulthood [4, 13]. Additionally, juvenile and adolescent onset CD has more frequent involvement of the small bowel, while adult CD is generally more likely to be colon-associated [15–19].

An altered immune response, together with genetic and environmental factors, may govern the timing of CD onset. There is a higher frequency of CD family history in patients diagnosed before the age of 20 relative to those diagnosed later in life, suggesting that genetic predisposition plays a role in early onset [13, 20]. Furthermore, immune mechanisms may be important in the pathogenesis of CD. For example, it has been shown in adult CD that higher levels of serum antibody reactivity towards increasing amount of microbiota in small intestine correlate with a greater frequency of complications [21, 22]. A similar observation has been made in a pediatric cohort [23, 24]. Furthermore, CD patients have been shown to have increased numbers of circulating Th17 lymphocytes and upregulation of IL17 transcriptional activity in intestinal mucosa [25]. This indicates that the progression of CD is characterized by an exacerbated Th17 response, which may explain the continuing nature of the disease.

Immune mechanisms are believed to play a role in the pathogenesis of adult and juvenile CD, with cytokines being essential to establish and maintain the immune response. However, little is known about differences in serum cytokine profiles between juvenile and adult CD cases. Therefore, we sought to determine whether the cytokine profiles differ. Potentially this information could suggest biomarkers related to the age of onset. We have found that all CD patients have upregulated serum CXCL10 levels, regardless of the age of diagnosis and the stage of the disease. Also, we have demonstrated upregulation of IL-1b and IL-6 in juvenile CD, with no changes detected in levels of GM-CSF and IFN-*γ*. In contrast, the adult CD serum profile was characterized by upregulation of GM-CSF and IFN-*γ*, as well as IL-6 and IL-1b, when compared to controls. It has been shown that GM-CSF and IFN-*γ* promote a Th1 immune response, while IL-6 is essential for activation of Th17 lymphocytes [26–28]. Therefore, this suggests that the mechanisms of pathogenesis differ in adult and juvenile CD patients.

## 2. Materials and Methods

### 2.1. Subjects

Clinical surplus serum samples from 64 patients diagnosed with CD were utilized in this study; 12 juvenile patients (6 male, 6 female; average age 14.0 ± 2.1) hospitalized in the Volga Region Federal Medical Research Center of the Ministry of Public Health (Nizhny Novgorod, Russia) and 52 adult patients (26 male, 26 female, average age 35.1 ± 13) hospitalized in the Department of Gastroenterology of Republican Clinical Hospital (Kazan, Russia). Diagnosis of CD was established in all patients based on clinical presentation and was verified by upper GI endoscopy.

Serum samples were collected from patients during both acute stage and remission. Additionally, a small number of samples (6) were collected from juvenile patients during their period of clinical improvement. Age and gender matched control samples were collected from 10 juvenile (5 male, 5 female) as well as from 23 adult (13 male, 10 female) controls. All serum samples were stored at −80°C until use. The study was approved by the Ethics Committee of Kazan State Medical University (protocol 23) and the Ethics Committee of Volga Region Federal Medical Research Center of the Ministry of Public Health Nizhny Novgorod (protocol 4). Informed consent was obtained from each study subject and legal guardian.

### 2.2. Cytokine Analysis

Serum cytokine levels were analyzed using single-plex sets for IL-1b, IL-2, IL-5, IL-4, IL-6, IL-8, IL-10, IL-12p40, GM-CSF, IFN-*γ*, CXCL10, and TNF-*α* (Bio-Rad, Hercules, CA, USA) following the manufacturer's instructions. Serum aliquots (50 *μ*L) were used for analysis, with a minimum of 50 beads acquired per analyte. Median fluorescence intensities were measured using a Luminex 200 analyzer. Data collected was analyzed with MasterPlex CT control software and MasterPlex QT analysis software (Hitachi Software, San Bruno, CA, USA). Standard curves for each analyte were generated using standards provided by the manufacturer.

### 2.3. Statistical Analysis

Statistical analysis was performed using the STATISTICA 7.0 Software Package (StatSoft, Tulsa, OK, USA). Data are presented as the median (25th–75th percentile range) for continuous variables. Differences between independent study groups were tested by nonparametric methods. The Kruskal-Wallis ANOVA by Ranks test was used for multiple independent samples. Pair comparisons were made by the Mann–Whitney *U* test. Differences were considered significant at *P* < 0.05.

## 3. Results

### 3.1. Serum Cytokine Activation in Juvenile Crohn's Disease Patients

Levels of 12 cytokines were analyzed in juvenile CD serum collected during both the acute stage and remission. Acute serum was characterized by upregulation of IL-1b, IL-6, CXCL10, and TNF-*α* levels compared to controls (Table 1). In contrast, no changes in IL-2, IL-5, IL-4, IL-10, IL-12p40, GM-CSF, and IFN-*γ* were found in acute juvenile patients compared to controls. Median values of IL-8 in acute serum were upregulated (1415.30 [997.1–2654.4] versus 2777.00 [611.0–7382.5]); however, differences were not statistically significant from controls.

During remission, serum levels of CXCL10 (*P* < 0.05) and IL-1b (*P* < 0.05) remained significantly upregulated in juvenile CD patients compared to controls (Table 1). The median value of IL-1b during remission was higher than that in acute patients (832.75 [481.9–1016.0] versus 550.00 [192.0–784.3]), suggesting an upward trend in cytokine production (*P* = 0.067). IL-6 and TNF-*α* serum levels were significantly higher during the acute stage but then declined in remission and remained comparable to controls. Similarly, in acute stage patients, median values of IL-8 were upregulated during remission (1415.30 [997.1–2654.4] versus 4 194.0 [411.9–7 220.9]); however, differences were not statistically significant from controls.

### 3.2. Analysis of Lymphocyte Profile in Juvenile CD Subgroups (Acute Stage and Remission)

Several leukocyte surface markers were examined including CD3 (lymphocytes), CD4 (T-helper (Th) lymphocytes), CD8 (cytotoxic T lymphocytes (CTL)), CD16 (natural killer (NK) cells), CD19 (B lymphocytes), and CD56 (NK cells) (Table 2). Increased numbers of CD16+CD56+ leukocytes were characteristic for acute stage juvenile CD compared to remission (0.26 ± 0.08 versus 0.14 ± 0.02 × 10^9^/L; *P* < 0.02) (Table 2). When the CD4/CD8 ratio was analyzed, two groups of acute stage patients could be identified having a ratio higher or lower than 2.0. Since cytokines play a central role in differentiation and proliferation of leukocyte CD4 and CD8 subsets, we analyzed serum cytokines in the CD4/CD8 subgroups of juvenile CD patients (Table 3). Acute stage patients with a CD4/CD8 ratio above 2.0 were characterized by upregulated serum levels of IL-1b, CXCL10, TNF-*α*, and IL-10. Additionally, high CD4/CD8 ratio patients had a greater IL-8 median value compared to those with a CD4/CD8 ratio below 2.0 (4161.50 [2956.0–8372.8] versus 1760.00 [257.4–6014.1]); however, these differences were not significant. Interestingly, CXCL10 levels were significantly higher in juvenile CD patients with a CD4/CD8 ratio below 2.0 than those with a CD4/CD8 ratio above 2.0.

### 3.3. Serum Cytokine Profile of Adult CD Patients

Serum cytokine profiles in adult CD patients depended on the stage (acute or remission) of the disease. For example, a subset of serum cytokines, including IL-1b, IL-6, GM-CSF, IFN-*γ*, and CXCL10, were upregulated in the acute stage of CD relative to controls (Table 4) and remained upregulated as in remission. However, serum levels of TNF-*α* and IL-10, unchanged in acute stage the disease, were downregulated during remission (Table 4). It should be noted that the IL-12 median value was 20-fold higher in adult CD patients in either acute stage or remission than controls, though not statistically significant. Although these differences were not significant, there was a strong trend towards upregulation in adult serum, both during the acute stage and the remission. Interestingly, in contrast, the serum level of IL-12 in juveniles in both the acute stage and the remission did not differ from controls. Further, in serum of acute stage, adults with CD both GM-CSF and IFN-*γ* were upregulated, whereas these cytokines remained unchanged in acute juvenile CD patients. This strongly suggests that several cytokines, including GM-CSF, IFN-*γ*, and IL-12, play a role in the pathogenesis of CD in adult patients.

## 4. Discussion

CD is a chronic inflammatory bowel disease affecting the integrity and function of the GI tract. The disease has a bimodal age distribution, with 20–25% of cases diagnosed early in life (childhood and adolescence), while 70% of cases are diagnosed in adulthood. CD incidence continues to increase especially in industrialized countries [29, 30]. Clinical symptoms of CD include abdominal pain, diarrhea, weight loss, and fever. In addition to the common GI symptoms, juvenile CD is often associated with failure to thrive that can include retarded growth, malnutrition, pubertal delay, and bone demineralization [31, 32].

Activation of innate and acquired immune responses are characteristic of CD. Studies using animal models have shown that CD pathogenesis is closely associated with a Th1 cytokine profile, while activation of the Th2 cytokines is more prevalent in the pathogenesis of ulcerative colitis [33, 34]. Activation of T cell-mediated immunity is linked to the development of epithelioid granulomas, which are pathognomonic to CD [35].

The serum cytokine profile of CD patients is also indicative of activation of a Th1 and a Th17 type immune response. For example, upregulation of IL-17A, IL-17F, IL-21, IL-22, IL-26, and CCL20 has been demonstrated in CD cases pointing to activation of Th17 type immunity [36]. Increased serum levels of TNF-*α* are a hallmark of CD suggesting a fundamental role for this cytokine in disease pathogenesis [37–39]. The role of cytokines in CD was supported by Prehn et al. who demonstrated enhanced production of IFN-*γ*, the key cytokine for activation of a Th1 type immune response, by mucosal T lymphocytes upon stimulation with TNF-*α* [40]. Therefore, these data suggest that cytokines play an important role in activating and maintaining the Th1 and Th17 type immune response, which is indicative of CD.

Although the role of cytokines in CD pathogenesis is well established, little is known about age differences in cytokine activation in CD patients. Juvenile CD is usually characterized by severe clinical manifestations, often including development of strictures or fistulae requiring surgical intervention, whereas adults frequently have a milder clinical presentation with a lower likelihood of developing complications [4, 13]. Therefore, we sought to determine whether the cytokine profiles in juvenile and adult CD differed. Serum levels of 12 cytokines were evaluated during both the acute stage and the remission in juvenile and adult CD patients. An interesting observation was that there was upregulation of CXCL10 cytokine in all patients regardless of age and stage of the disease. Although increased CXCL10 serum levels and enhanced expression of CXCL10 in inflammatory bowel tissue has previously been shown in adult CD, here we present the first evidence for upregulation of this chemokine in the serum from juvenile CD cases. CXCL10 is a chemokine known to exclusively recruit activated T lymphocytes and NK cells [41–43]. Additionally, it has been demonstrated that CXCL10 targets Th1 lymphocytes [42, 43]. The essential role of CXCL10 in the gut lymphocyte infiltration and inflammation has been established by Grip and Janciauskiene [44]. These authors demonstrated that reduced plasma levels of CXCL10 correlate with decreased level of the prominent inflammation marker, C reactive protein. In another study, Hyun et al. demonstrated that a reduction in CXCL10 serum levels impairs activation and reduces recruitment of Th1 lymphocytes into the gut mucosa and lymphoid tissue [45]. Therefore, our data support the notion that upregulation of CXCL10 can facilitate Th1 lymphocyte infiltration and perpetuate epithelial inflammation in the gut. Furthermore, increased serum levels of CXCL10 were found in all patients regardless of the stage of the disease, suggesting chronic Th1 lymphocyte activation, which is ongoing even during clinical remission.

An interesting observation regarding serum IL-8 levels in juvenile and adult CD patients was that, although not significant, the serum level of IL-8 was higher (1.9 and 2.9 times) in acute and remission juvenile CD compared to controls. In contrast, serum IL8 levels in adult CD remained similar to age-matched controls. IL-8 is a pleotropic cytokine, known as the prototype neutrophil attractant [46], which also activates neutrophils by upregulating phagocytosis and respiratory burst [47]. Since inflamed tissue is often infiltrated by activated neutrophils [48], this suggests a contribution of IL-8 to both local and systemic inflammation. Although high circulating IL-8 is characteristic of inflammation, a lack of change in serum IL-8 in some cases of gastroduodenitis has been documented [49]. Further investigation demonstrated that although IL-8 levels in serum were unchanged, there were increased levels of IL-8 transcripts within intestinal tissue [50, 51], suggesting local production of IL-8. Therefore, this suggests that lack of changes in IL-8 in serum may not always reflect ongoing in situ inflammation. Therefore, we propose that in juvenile CD that high serum IL-8 may reflect systemic inflammation, whereas, in adult CD, the lack of changes in circulating IL-8 level indicates that inflammation could be restricted to bowel tissue.

Another finding was that the serum level of TNF-*α* was significantly upregulated in juvenile CD compared to controls, while it remained unchanged in adult cases. These data suggest that juvenile CD, relative to adult, is characterized by a stronger inflammatory milieu in the gut. Additionally, increased serum IL-6 was detected all CD cases, regardless of patient's age, relative to controls. IL-6 is a B cell differentiating factor [52] that, when combined with TGF*β*, promotes the differentiation of Th17 lymphocytes [53]. Moreover, IL-6 is considered an essential cytokine for Th17 differentiation of naive T cells [54]. Th17 lymphocytes are believed to be pathogenic and are often associated with the severe tissue damage and development of autoimmunity [55]. The mechanisms of Th17-associated tissue damage are linked to a breach in the integrity of the intestinal epithelium due to increased production of inflammatory cytokines and matrix metalloproteinases, as well as inhibition of proliferation of human gut epithelial cells [56, 57]. Interestingly, juvenile CD is characterized by upregulation of the IL-6, while levels of IFN-*γ* were unaffected. However, the opposite pattern of cytokine activation was found in adult CD, where only IFN-*γ* levels were significantly upregulated. IFN-*γ* is secreted by CTL and often used as a marker of the Th1 type immune response [58]. The Th1 (IL12-IFN-*γ*) and Th17 (IL-6-IL-17) pathways seem to be mutually exclusive, with IFN-*γ* and IL-17 acting as reciprocal inhibitory cytokines [59]. However, a subpopulation of Th17 lymphocytes producing IL-17 and IFN-*γ* was identified in CD gut tissue by Annunziato et al. [60]. The authors suggested that IL-17/IFN-*γ* positive Th17 are important for CD pathogenesis, as these cells were able to support B cell proliferation, had low cytotoxic activity, and were poorly responsive to autologous regulatory T cells. Therefore, we hypothesize that the Th17 response is characteristic for juvenile and adult CD. However, upregulation of IL-17 and IFN-*γ* in adult CD suggests that there are more complex mechanisms of pathogenesis relative to juvenile CD. We propose that the Th17 (IL-17/IFN-*γ*), or a combination of Th17 and Th1 immune responses, play a leading role in pathogenesis of adult CD. Studies using tissue biopsies from juvenile and adult CD will help to better understand the role of these Th subsets in CD pathogenesis.

Another finding was upregulation of GM-CSF in adult CD patients. It has been suggested that GM-CSF can alter the course of intestinal inflammation. For example, Däbritz et al. have shown that GM-CSF activated macrophages protect mice from T cell-induced colitis [61]. These authors suggested that GM-CSF-activated macrophages facilitate differentiation of Th2 lymphocytes by upregulating production of IL-4, IL-10, and IL-13 in lamina propria resident leukocytes, which may decrease local inflammation, improve bacterial clearance, and promote healing. In another study, Gathungu et al. have shown that the presence of anti-GM-CSF antibody can serve as marker for the severity of CD [62]. Further, these authors demonstrated that elevated anti-GM-CSF antibody levels were associated with intestinal stricture, penetration, and resection in CD patients. A similar observation was published by Gathungu et al., where CD patients with elevated anti-GM-CSF antibody exhibited increased bowel permeability as compared to those with lower levels of this antibody [62]. It has been suggested that GM-CSF neutralization promotes ileac disease by modulating expression of CCL25 by intestinal epithelium and CCR9 by T lymphocytes via Nod2-dependent and -independent pathways [62]. Therefore, increased GM-CSF levels in adult CD may contribute to inhibition of bowel inflammation and prevention of tissue damage.

Adult CD was characterized by upregulation of serum GM-CSF and IFN-*γ*, a combination with strong anti-inflammatory potential. For example, it has been shown that treatment of an LPS challenge with GM-CSF and IFN-*γ* inhibited an inflammatory response by lowering neutrophil counts and increasing the number of suppressive neutrophils (CD16 (bright)/CD62L (dim)) [63] in healthy volunteers [64]. The GM-CSF/IFN-*γ* combination may have a profound effect on leukocyte polarization. Both cytokines play a key role in developing M1 type macrophages, which are associated with Th1 type immune response [65]. M1 macrophages exhibit both strong microbiocidal activity and cell proliferation inhibitory capacity [66]. Therefore, increased serum GM-CSF and IFN-*γ* may reflect activation of Th1 type immune response in adult CD.

## 5. Conclusion

Our data suggest that juvenile and adult CD are characterized by unique serum cytokine profiles, which may reflect age-dependent difference in disease pathogenesis.

## Figures and Tables

**Table 1:** Serum cytokine activation in juvenile CD (median [25th–75th percentile]).

Cytokine (pg/mL)	Controls (*n* = 10)	Acute patients (*n* = 25)	Remission patients (*n* = 12)
IL-1b	107,7 [16,4–340,8]	550,0 [192,0–784,3]^*∗*^	832,75 [481,9–1 016,0]^*∗*^
IL-2	2,3 [1,5–3.0]	4,3 [3,2–5,4]	5,5 [4,1–7,7]
IL-5	9,0 [3,5–11,8]	6,4 [3,4–16,0]	8,0 [4,5–15,6]
IL-6	23,5 [17,2–27,3]	51,8 [22,8–67,3]^*∗*^	20,3 [12,9–25,3]^*∗∗*^
IL-8	1 415,3 [997,1–2 625,4]	2 777,0 [611,0–7 382,5]	4 194,0 [411,9–7 220,9]
IL-12	203,2 [120,3–280,0]	217,5 [137,5–519,5]	225,8 [69,8–286,1]
GM-CSF	30,4 [13,5–38,0]	32,0 [10,2–45,3]	35,3 [21,6–50,0]
IFN-*γ*	2,5 [1,8–3,2]	3,8 [3,2–4,8]	2,8 [2,2–4,3]
CXCL10	384,9 [265,3–435,4]	3 660,0 [2 809,5–4 457,5]^*∗*^	3 685,5 [2 663,5–5 124,0]^*∗*^
TNF-*α*	4,6 [3,3–6,2]	8,5 [5,3–12,0]^*∗*^	5,0 [3,4–8,6]
IL-4	8,5 [6,3–9,1]	8,7 [7,4–11,7]	7,1 [6,2–8,4]
IL-10	23,9 [17,6–32,4]	22,0 [16,0–60,0]	11,8 [5,9–20,3]^*∗∗*^

^*∗*^ *P* < 0.05 versus controls (Mann–Whitney test).

^*∗∗*^ *P* < 0.05 versus acute patients (Mann–Whitney test).

**Table 2:** Changes in the peripheral lymphocyte population in juvenal CD.

Lymphocytes (10^9^/L)	Control (*n* = 10)	Acute patients (*n* = 25)	Remission patients (*n* = 12)
CD3+	1.91 ± 0.5	1.34 ± 0.4	1.22 ± 0.3
CD8+	0.73 ± 0.2	0.52 ± 0.2	0.56 ± 0.1
CD4+	1.93 ± 0.3	0.82 ± 0.1	0.74 ± 0.2
CD16+CD56+	0.12 ± 0.1	0.26 ± 0.08^*∗*^	0.14 ± 0.02^*∗∗*^
CD19+	0.71 ± 0.3	0.34 ± 0.2	0.25 ± 0.2

^*∗*^ *P* < 0.05 between patients and control.

^*∗∗*^ *P* < 0.02 between acute and remission CD.

**Table 3:** Serum cytokine activation in CD4/CD8 ratio subgroups of juvenile CD (median [25th–75th percentile]).

Cytokine (pg/mL)	Controls (*n* = 10)	Acute patients, CD4/CD8 < 2 (*n* = 18)	Acute patients, CD4/CD8 > 2 (*n* = 7)
IL-1b	107,7 [16,4–340,8]	392,7 [131,6–807,7]	567,5 [444,5–650,0]^*∗*^
IL-2	2,3 [1,5–3.0]	4,4 [3,4–6,2]^*∗*^	3,2 [2,8–3,9]
IL-5	9,0 [3,5–11,8]	5,9 [2,7–13,6]	15,3 [4,7–22,2]
IL-6	23,5 [17,2–27,3]	54,3 [20,32–66,7]	27,8 [25,2–99,1]
IL-8	1 415,3 [997,1–2 625,4]	1760,0 [257,4–6014,1]	4161,5 [2956,0–8372,8]
IL-12	203,2 [120,3–280,0]	204,5 [144,1–426,5]	257,0 [171,8–885,3]
GM-CSF	30,4 [13,5–38,0]	33,0 [13,5–45,5]	29,5 [9,5–33,5]
IFN-*γ*	2,5 [1,8–3,2]	4,15 [3,2–5,2]^*∗*^	3,4 [2,5–4,4]
CXCL10	384,9 [265,3–435,4]	4089,5 [2835,8–4597,8]^*∗*^	2925,0 [2448,5–3426,0]^*∗*,*∗∗*^
TNF-*α*	4,6 [3,3–6,2]	7,4 [5,3–9,4]^*∗*^	12,0 [11,5–13,0]^*∗*,*∗∗*^
IL-4	8,5 [6,3–9,1]	8,9 [7,4–11,3]	8,7 [7,6–11,3]
IL-10	23,9 [17,6–32,4]	16,8 [14,3–28,0]	60,0 [32,3–94,5]^*∗*,*∗∗*^

^*∗*^ *P* < 0.05 versus controls (Mann–Whitney test).

^*∗∗*^ *P* < 0.05 versus acute patients with CD4/CD8 ratio < 2.0 (Mann–Whitney test).

**Table 4:** Serum cytokine activation in adult CD (median [25th–75th percentile]).

Cytokine (pg/mL)	Controls (*n* = 7)	Acute patients (*n* = 36)	Remission patients (*n* = 16)
IL-1b	17.4 [11.3–26.1]	47.1 [32.2–87.2]^*∗*^	53.2 [31.0–98.0]^*∗*^
IL-2	110.0 [92.0–141.0]	102.0 [89.0–131.7]	93.0 [52.5–112.5]
IL-4	45.3 [41.6–77.2]	41.0 [34.8–47.8]	56.8 [45.4–68.9]
IL-5	15.2 [11.2–17.2]	12.8 [11.2–16.4]	14.2 [11.2–22.4]
IL-6	120.3 [110.2–128.3]	177.4 [130.6–296.7]^*∗*^	307.8 [174.0–636.2]^*∗*^
IL-8	207.5 [114.4–289.4]	293.3 [163.5–593.9]	236.3 [153.2–625.5]
IL-10	238.4 [166.4–273.1]	258.3 [196.0–291.3]	167.3 [153.5–180.7]^*∗∗*^
IL-12	18.0 [18.0–415.2]	475.7 [18.0–1076.5]	487.9 [18.0–1 127.1]
CXCL10	6 910.5 [4 729.3–10 178.0]	21 111.7 [17 153.1–27 546.8]^*∗*^	20 009.8 [18 836.5–25 123.4]^*∗*^
GM-CSF	80.2 [45.0–113.7]	199.2 [70.0–470.0]^*∗*^	120.7 [90.9–494.4]
IFN-*γ*	2 967.0 [2 344.5–3 675.6]	6 750.4 [4 806.5–11 050.0]^*∗*^	6 846.2 [4 054.3–11 456.4]^*∗*^
TNF-*α*	179.2 [143.6–196.4]	193.0 [179.0–211.3]	113.3 [76.2–171.0]^*∗∗*^

^*∗*^ *P* < 0.05 versus controls (Mann–Whitney test).

^*∗∗*^ *P* < 0.05 versus acute patients (Mann–Whitney test).
